# IL-6 expression predicts treatment response and outcome in squamous cell carcinoma of the esophagus

**DOI:** 10.1186/1476-4598-12-26

**Published:** 2013-04-05

**Authors:** Miao-Fen Chen, Ping-Tsung Chen, Ming Shian Lu, Paul Yang Lin, Wen-Cheng Chen, Kuan-Der Lee

**Affiliations:** 1 Department of Radiation Oncology, Chang Gung Memorial Hospital at Chiayi, Chiayi, Taiwan; 2 Chang Gung University, College of Medicine, Taoyuan, Taiwan; 3 Department of Hematology and Oncology, Chang Gung Memorial Hospital at Chiayi, Chiayi, Taiwan; 4 Graduate Institute of Clinical Medical Sciences, College of Medicine, Chang Gung University, Taoyuan, Taiwan; 5 Department of Thoracic & Cardiovascular Surgery, Chang Gung Memorial Hospital, Chiayi, Taiwan; 6 Department of Pathology, Chang Gung Memorial Hospital at Chiayi, Chiayi, Taiwan

**Keywords:** IL-6, Esophageal squamous cell carcinoma, Prognosis

## Abstract

**Background:**

The identification of potential tumor markers can improve therapeutic planning and patient management. The aim of this study was to highlight the significance of IL-6 in esophageal squamous cell carcinoma (SCC).

**Methods:**

We retrospectively analyzed the clinical outcomes of 173 patients with esophageal SCC, and examined the correlation between IL-6 levels and clinical outcomes in esophageal cancer patients. Furthermore, the human esophageal SCC cell line CE81T was selected for cellular and animal experiments to investigate changes in tumor behavior and treatment response after manipulation of IL-6 expression.

**Results:**

In clinical outcome analysis, positive IL-6 staining and poor treatment response was significantly associated with shorter survival. Furthermore, the frequency of IL-6 immunoreactivity was significantly higher in esophageal cancer specimens than in non-malignant epithelium, and this staining was positively linked to the development of distant metastasis (*p* = 0.0003) and lower treatment response rates (*p* = 0.0001).By ELISA analysis, IL-6 serum levels were significantly elevated in patients developing disease failure.When IL-6 expression was inhibited, aggressive tumor behavior and radiation resistance could be overcome *in vitro* and *in vivo*. The underlying changes included increased cell death, less epithelial-mesenchymal transition and attenuated STAT3 activation. IL-6 inhibition was also associated with attenuated angiogenesis in tumor-bearing mice.

**Conclusions:**

IL-6 was significantly associated with poor prognosis in patients with esophageal cancer. Targeting this cytokine could be a promising strategy for treatment of esophageal cancer, as evidenced by inhibition of aggressive tumor behavior and treatment resistance.

## Background

Esophageal cancer, an aggressive upper gastrointestinal malignancy, generally presents as a locally advanced disease and requires a multimodal concept. Despite improvements in its detection and management, the prognosis in patients with esophageal cancer remains poor, with a 5-year survival of 15–34% [1,2]. Most patients who undergo curative treatment for esophageal cancer will eventually relapse and die as a result of their disease. Neoadjuvant chemoradiotherapy (CCRT) can increase the chance of R0 resection, and responders will have a survival benefit [3]. Patient selection is important to guide multidisciplinary therapy. The identification of potential molecular markers to predict response to CCRT and recurrence is important for the effective management and prognosis of esophageal cancer.

Proinflammatory cytokine may contribute to tumor progression by stimulating angiogenesis, invasion and metastasis [4,5] IL-6 is a pleiotropic cytokine that is capable of modulating diverse cell functions such as inflammatory reactions, and is a major activator of the JAK/STAT3 and PI3K/AKT signaling pathways [6]. IL-6 signaling has been implicated in the regulation of tumor growth and metastatic spread in different cancers [7-9]. Moreover, increased IL-6 serum levels were reported to be associated with metastasis and poor prognosis in prostate, ovarian and gastrointestinal cancers [10-12]. Although evidence suggests that IL-6 is a critical factor in a variety of malignancies [11,13,14], how IL-6 modulates the biological activities of esophageal carcinoma cells and how it is associated with the prognosis of esophageal cancer remains unclear. There are two distinct histological types of esophageal cancer: squamous cell carcinoma (SCC) and adenocarcinoma. There are marked differences between these carcinomas in incidence, natural history and treatment outcomes [15]. The majority of esophageal SCC cases occur in Asia, with the predominant type in Taiwan being SCC [16]. We therefore investigated the role of IL-6 in esophageal SCC *in vitro* and *in vivo*, and examined the correlation between IL-6 levels and clinical outcomes in esophageal cancer patients.

## Materials and methods

### Patient characteristics

The Institutional Review Board of Chang Gung Memorial Hospital approved the present study (Permit Number: 96-1693B). The written consents were signed by the patients for their specimen and information to be stored in the hospital and used for research. Patients who did not comply with the treatment regimen and those who received surgery alone for early-stage esophageal cancer were excluded from the study. A total of 173 patients with esophageal SCC who completed curative treatment were enrolled in the study. The curative treatment for esophageal cancer included neoadjuvant CCRT combined with surgery or definitive CCRT according to the guidelines proposed by oncology team at our hospital. On completion of neoadjuvant CCRT, patients underwent a repeat CT scan and endoscopic examination to determine the response to treatment. If the tumor was considered resectable, patients underwent surgery for the residual tumor. Pathologic complete response (CR) was defined as absence of residual invasive tumor in the surgical specimen in patients undergoing surgical intervention. For patients who refused surgery or in whom it was contraindicated, a second round of CCRT was administered, comprising two courses of chemotherapy and radiotherapy of a total of 60–63 Gy. Specimens collected from the 173 patients at diagnosis were subjected to immunochemical analysis. The main end points were overall survival (OS), disease-free survival (DFS) and response to neoadjuvant therapy. Survival probability was analyzed statistically using the Kaplan–Meier method. The significance of between-group differences was assessed using the log-rank test. Multivariate analyses were performed using a Cox regression model for survival.

### Immunohistochemical staining (IHC)

Formalin-fixed, paraffin-embedded tissues from 173 patients with esophageal cancer were subjected to IHC staining. Dissected esophageal cancer specimens from 20 of these patients were converted into tissue microarray (TMA) blocks using an AutoTiss 1000 arrayer (Ever BioTechnology, Canada). The TMA block contained esophageal SCCs and the adjacent non-malignant epithelium. The quality of the TMA slides was confirmed by the pathologist using hematoxylin- and eosin-stained slides. The IHC data for the specimens were assessed using the semi-quantitative immunoreactive score (IRS). The criterion for positive staining was an IRS score of ≥2. The details were described in Additional file 1: Supplementary methods.

### Cell culture and reagents

The human esophageal cancer cell line CE81T, derived from a well-differentiated esophageal SCC, was obtained from the Bioresource Collection and Research Center (BCRC),and cultured in Dulbecco’s modified Eagle’s medium supplemented with 10% fetal bovine serum. The IL-6-neutralizing antibody, STAT3 siRNA and the JAK inhibitor AG490 were purchased from R&D Systems (Minneapolis, MN) and Sigma (St. Louis, MO), respectively. The IL-6-GFP silencing vector (human IL6 shRNA constructs in retroviral GFP vector) and GFP-control vector (Non-effective scrambled shRNA cassette in retroviral GFP vector) were purchased from Origene Technologies, Inc. (Rockville, MD). Transfection in CE81T was carried out by Lipofectamine™ 2000 (Invitrogen) according to the manufacturer’s instructions. Stable cancer cells were generated by transfecting CE81T cells with either the IL-6 silencing vectors or control vectors, followed by selection with puromycin for 4 weeks.

### Cell growth

To measure cell growth, 1×10^4^ cells per well were plated into 6-well dishes. At the indicated time-points, cells were trypsinized, collected and surviving cells counted using Trypan blue exclusion, from which the survival curves for CE81T transfectants ( wild type, with control vectors, and with IL-6 silencing vectors) were established.

### Immunoblotting

To determine the *in vitro* effects of STAT3 siRNA, proteins were extracted from cells transfected with STAT3 siRNA for 72 h. To determine the *in vitro* effects of the JAK inhibitor and the anti-IL-6 antibody, proteins were extracted from cells incubated in the presence of 50 μM AG490 or 5 μg/ml IL-6 neutralizing antibodies for 24 h. The details were described in Additional file 1: Supplementary methods.

### Immunofluorescence staining (IF) and Statistical analysis

The details were described in Additional file 1: Supplementary methods.

### Enzyme-linked immunosorbent assay (ELISA) analysis of IL-6 level

We examined the serum level of IL-6 from 71 patients among subjects enrolled with esophageal cancer in the present study. For serum specimen, five milliliters of peripheral blood were drawn from each patient. Moreover, IL-6 levels in cellular supertnant and murine serum were tested. Levels of IL-6 in samples were analyzed using an IL-6 Quantikine ELISA Kit (R&D system). The details were described in Additional file 1: Supplementary methods.

### Tumor xenografts

This study was carried out in strict accordance with the recommendations in the Guide for the Care and Use of Laboratory Animals as promulgated by the Institutes of Laboratory Animal Resources, National Research Council, U.S.A. The protocol was approved by the Committee on the Ethics of Animal Experiments of Chang Gung Memorial Hospital (Permit Number: 2012080903). Eight-week-old male athymic nude mice were used and all animal experiments conformed to the protocols approved by the experimental animal committee of our hospital. CE81T cancer cell transfectants (1 × 10^6^ per implantation, five animals per group) were subcutaneously implanted in the dorsal gluteal region. To determine *in vivo* radiosensitivity, local irradiation (15 Gy) was performed when the tumor volume reached 500 mm^3^. Radiosensitivity was indicated by a growth delay (*i*.*e*., the time required for the tumor to recover its previous volume after irradiation). Duplicate experiments were performed for growth delay analyses. The details were described in Additional file 1: Supplementary methods.

## Results

### Level of IL-6 in esophageal SCC

**Figure 1:**
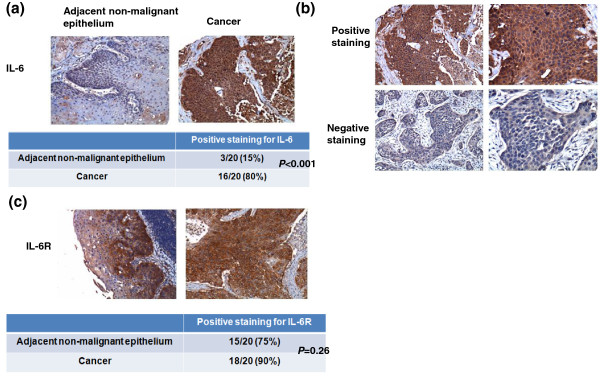
**IL-6 levels in esophageal SCC. a**. Representative IHC staining with an anti-IL-6 antibody of esophageal cancer and adjacent non-malignant epithelium from TMA blocks. **b**. IHC staining with an anti-IL-6 antibody of human esophageal cancer specimens. Images of representative slides are shown at magnifications of × 100 (left panel) and × 200 (right panel). **c**. Representative images of IHC staining with an anti-IL-6R antibody of esophageal cancer and adjacent non-malignant epithelium from TMA blocks.

The IHC data for TMA slides demonstrated that IL-6 was overexpressed in tumor tissues compared to adjacent non-malignant epithelial tissues (Figure 1a). Figure 1b showed the representative slides of positive staining and negative staining with anti-IL-6 antibody for human esophageal cancer specimens. Of the 173 esophageal cancer tissues, 88 (51%) showed positive IL-6 immunoreactivity (41% (45/109) in ≤ T3 *versus* 67% (43/64) in T4, *P* = 0.001). Furthermore, there was a positive correlation between IL-6 overexpression and cancers developing loco-regional failure or distant metastasis (Table 1). The biological activities of IL-6 are mediated through binding to a membrane-bound or soluble form of IL-6 receptor. By IHC analysis of TMA slides, IL-6 receptor (IL-6R) was expressed in both tumor tissues and adjacent non-malignant epithelial tissues (Figure 1c).

**Table 1:** Clinico-pathological characteristics of esophageal cancer patients for immunohistochemical investigation

	**No. of patients**		
	**IHC-IL-6(-)**	**IHC- IL-6 (+)**	***p *value**
**patients**	85	88	
**Age**			
> = 56y/0	43	44	0.938
<56y/o	42	44	
**Location**			
Upper third	22	26	0.498
Middle third	48	42	
Lower third	15	20	
**Tumor stage**			0.041*
I-II	28	17	
III-IV	57	71	
**LN metastasis**			0.246
negative	27	21	
positive	58	67	
**P-stat3 staining**			<0.0001*
negative	71	20	
positive	14	68	
**Response to Neoadjuvant Tx**			0.0001*
Response	69	47	
Non- response	16	41	
**Surgery s/p Neoadjuvant Tx**			0.145
Yes	31	24	
No	54	64	
**Local-regional Recurrence /persistent**			<0.0001*
No	44	16	
Yes	41	72	
**Distant metastasis**			0.0003*
negative	58	36	
positive	27	52	

Abbreviations: Neoadjuvant Tx = neoadjuvant chemoradiotherapy.

### Role of IL-6 in tumor growth

**Figure 2:**
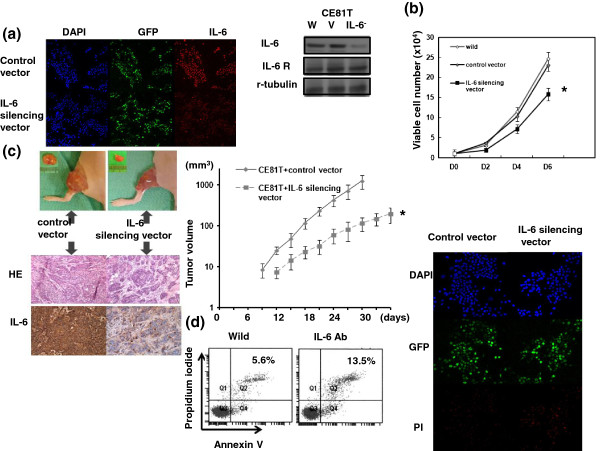
**Role of IL-6 in tumor growth. a**. Effect of an IL-6-GFP silencing vector on the level of IL-6 in CE81T, as assessed by immunofluorescence and immunoblotting. The results of representative slides are shown. The IL-6-GFP silencing vector as compared with that with control-GFP vector significantly reduced IL-6 levels. **b**. Effect of IL-6 on the proliferation of CE81T cancer cells as determined by viable cell counting. The same number of cells (10^4^) were plated in each plate on day 0 and allowed to grow in their respective cultures. We counted the number of viable cells after incubation for 2, 4 and 6 days. The Y axis represents the viable cell number. Point, means of three separate experiments; bars, SD. *, *p* < 0.05. **c**. Effect of IL-6 inhibition on tumor xenograft growth. Data represent the means ± SD of three independent experiments. *, *p* < 0.05. IL-6 expression was also evaluated by IHC staining of xenografts. Representative slides are shown. **d**. Effect of IL-6 inhibition on cell death, as assessed by FACS and immunofluorescence staining. The results of representative slides are shown.

As demonstrated in Figure 2a, the IL-6 silencing vector significantly inhibited IL-6 expression in CE81T cells. By viable cell counting over 6 days and observation of xenograft tumors, the IL-6 silencing vector significantly inhibited tumor growth *in vitro* (Figure 2b) and *in vivo* (Figure 2c). Figure 2d showed that the cell death rate increased from 5.6 ± 1.2% to 13.5 ± 1.8% in CE81T cells after treatment with an IL-6-neutralizing antibody (as measured by flow cytometry) and by the IL-6 silencing vector (propidium iodide staining).

### Role of IL-6 in tumor invasion and underlying mechanisms

**Figure 3:**
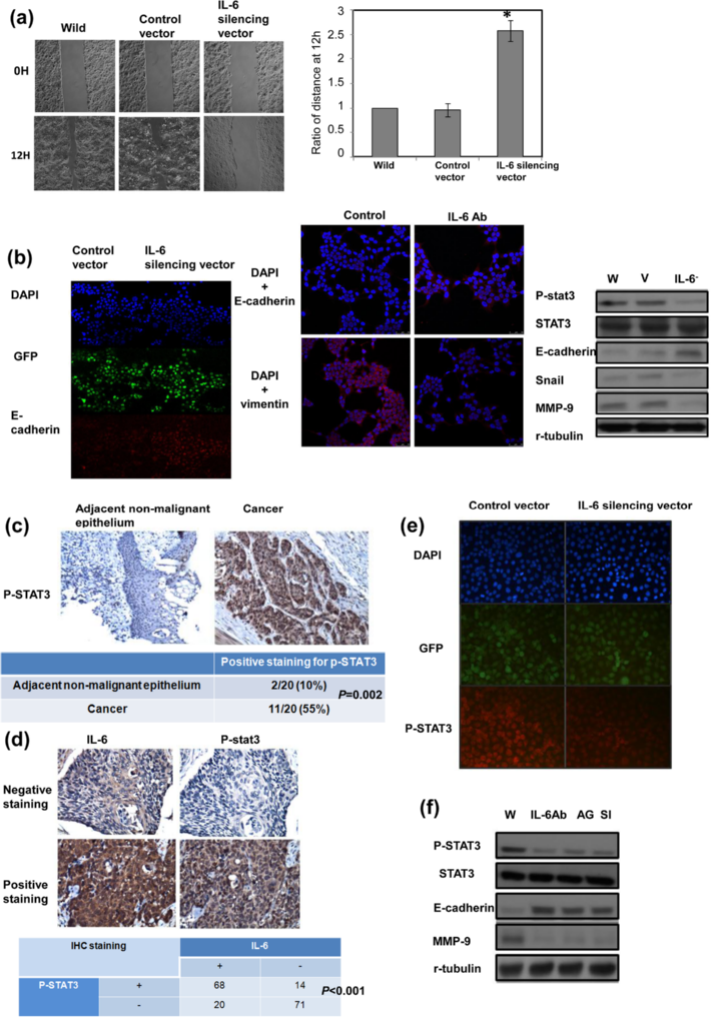
**Role of IL-6 in aggressive tumor behavior and EMT changes. a**. Effect of IL-6 inhibition on the migration ability of esophageal cancer cells. Plates were photographed at the times indicated. Representative slides and quantitative data (y-axis shows the relative ratio, normalized to the distance under control conditions) are shown. **b**. Effect of IL-6 inhibition on EMT-associated proteins. The changes of E-cadherin and vimentin expression were evaluated by immunofluorescence, and the results of representative slides are shown. In addition, changes in the levels of EMT-associated proteins were evaluated by immunoblotting (W, wild-type cells; V, cells transfected with the control vector; IL-6^-^, cells transfected with the IL-6 silencing vector). **c**. Representative IHC staining with an anti-p-STAT3 antibody in esophageal cancer and adjacent non-malignant epithelium on slides from TMA blocks. **d**. P-STAT3 levels correlate positively with IL-6 levels in human esophageal cancer specimens (*p* < 0.001). Representative images of positive IL-6 and p-STAT3 staining on slides from a selected tumor specimen, and representative negative staining for IL-6 and p-STAT3 on slides from another tumor specimen, are shown. **e**. Effect of the IL-6 silencing vector on STAT3 activation, as examined by immunofluorescence analysis. **f**. Effect of inhibited IL-6 signaling on STAT3 activation and EMT-related protein levels, as determined by immunoblotting (W, proteins were extracted from cells under control condition; IL-6Ab, proteins were extracted from cells incubated in the presence of 5 μg/ml IL-6 neutralizing antibodies for 24 h; AG, proteins were extracted from cells incubated in the presence of 50 μM AG490 for 24 h; SI, proteins were extracted from cells 72 h after transfection with STAT3 siRNA).

There was a positive link between IL-6 and tumor stage and disease failure in patients with esophageal cancer (Table 1). Furthermore, as demonstrated using migration scratch assays, IL-6 silencing vector attenuated the invasive capacity of esophageal cancer cells (Figure 3a). Epithelial-mesenchymal transition (EMT) is a key event in invasiveness [17]. As shown in Figure 3b, the IL-6 silencing vector reversed EMT changes, with increased E-cadherin expressions, and decreased matrix metalloproteinase (MMP)-9, vimentin and Snail, a repressor of E-cadherin expression. The activation of STAT3 signaling was reported to play important roles in the induction of aggressive tumor behavior and EMT changes in cancer [18], including cancers in the upper aerodigestive tract [8,19]. As shown in Table 1 and Figure 3c–d, positive staining for p-STAT3 was evident in 47% of the 173 cancer specimens, and a significant positive correlation was found in cancer specimen that expressed IL-6 and p-STAT3. As determined by immunofluorescence and immunoblotting analysis (Figure 3e-f), IL-6 inhibition significantly attenuated the activation of STAT3 *in vitro*. Moreover, when STAT3 signaling was inhibited by STAT3 siRNA or the JAK inhibitor AG490, the decreases in EMT- related protein levels were comparable to those induced by the IL-6-neutralizing antibody (Figure 3f). Therefore, it appears that altered STAT3 activation and subsequent EMT might, at least in part, be responsible for the aggressive tumor behavior in IL-6-positive esophageal cancer.

### Effects of circulating IL-6 on tumor aggressiveness

**Figure 4:**
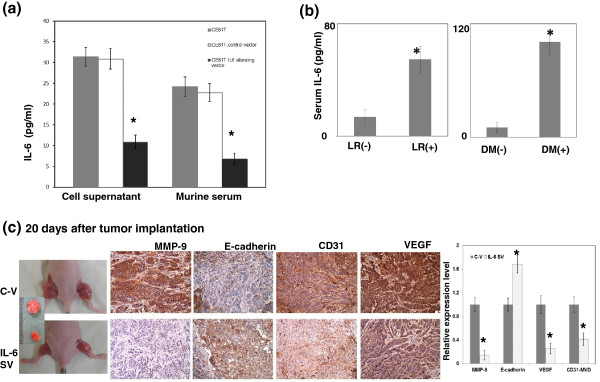
**Effects of circulating IL-6 on tumor aggressiveness. a**. The levels of IL-6 in cell supernatant and serum of mice bearing tumors with or without IL-6 silencing vectors were examined by ELISA. Columns, means of 3 separate experiments; bars, SD. *, *P* < 0.05. **b**. Serum IL-6 levels of patients were examined by ELISA analysis. Columns are the means of specimen; Bars, SD; *, *P  <*  0.05. (LR = Local-regional Recurrence /persistent; DM = distant metastasis). **c**. IHC using MMP-9, E-cadherin, CD31, and VEGF staining in tumors 20 days after implantation. IL-6 silencing vectors were significantly reduced by the angiogenesis and EMT-related changes as compared with control-GFP vector. The Y-axis represents the ratio normalized by the value of target protein in tumor transfected with control vectors. Columns, means of 3 separate experiments; bars, SD. *, *P* < 0.05 (C-V, cells transfected with the control vector; IL-6 SV, cells transfected with the IL-6 silencing vector).

We used ELISA assay to test the level of IL-6 in the supernatant of cell culture and the serum of mice bearing tumors. As shown in Figure 4a, IL-6 silencing vector clearly attenuated IL-6 secretion. Moreover, the serum levels of IL-6 were examined by ELISA. The mean IL-6 levels in serum samples from 71 patients with esophageal cancer were 39 ±  7.7 pg/mL. Serum IL-6 levels were significantly elevated in patients with local-regional failure or developing distant metastasis compared to those in patients with disease control (Figure 4b). Table 2 also showed that IL-6 serum levels were significantly correlated with a greater probability of developing local-regional failure and distant metastasis. In addition to EMT, angiogenesis is one of the mechanisms that promote tumor progression. The most prominent and best-characterized pro-angiogenic pathway is vascular endothelial growth factor (VEGF) signaling [20], and CD31-mediated endothelial cell-cell interactions are involved in angiogenesis [21]. Therefore, the vascular network within the tumor was measured by VEGF staining and microvascular density (MVD) analysis after CD31 staining. When esophageal cancer cells with control vectors and those with IL-6 silencing vectors were subcutaneously implanted into mice, we found that the growth inhibiting effect induced by IL-6 silencing vector associated with lower expression levels of EMT- and angiogenesis-related factor (Figure 4c). Accordingly, we suggested that circulating IL-6 plays a role in tumor promotion, and the induction of angiogenesis and EMT might be the underling mechanisms.

**Table 2:** Significance of IL-6 expression stratified according to the occurrence of local- regional failure (LR) or distant metastasis (DM) in 71 patients

		**IL-6 serum level**		
		**<mean**	**> = mean**	
**LR**	**+**	12	32	*P = 0.001*
	**-**	18	9	
**DM**	**+**	10	24	*P = 0.035*
	**-**	20	17	

LR = Local-regional Recurrence /persistent; DM = distant metastasis.

### IL-6 correlates with treatment response in esophageal SCC

**Figure 5:**
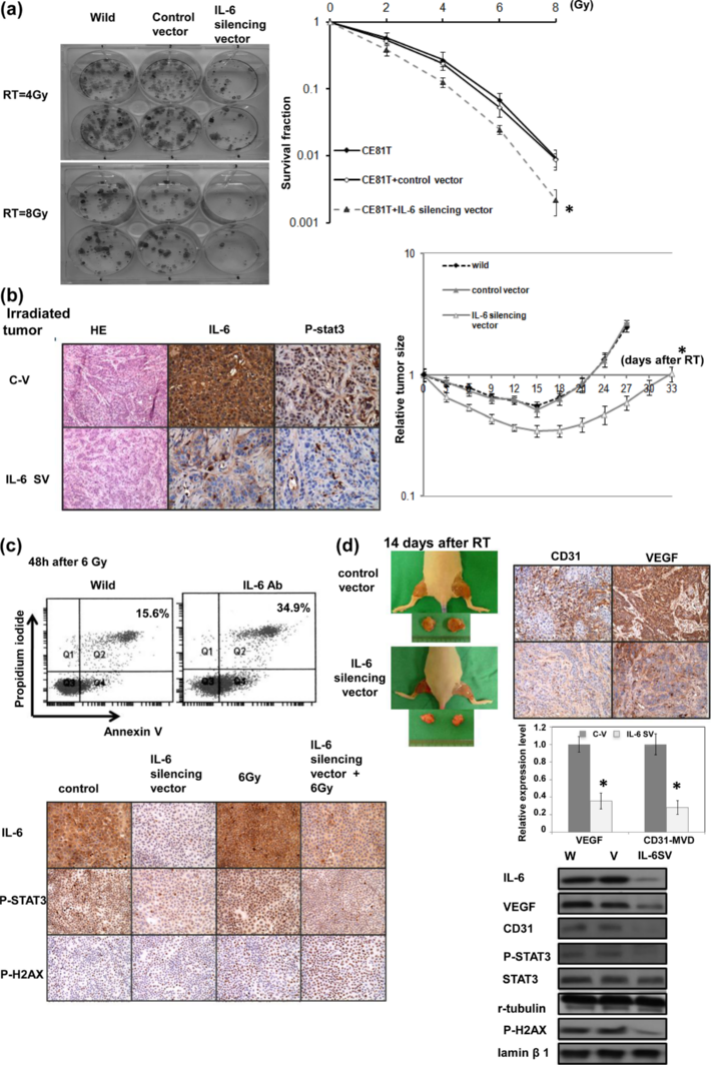
**Effects of IL-6 on radiation responses. a**. Cells were irradiated with 0, 2, 4, 6, and 8 Gy, and survival curves were constructed using colony-forming assay data. Data represent the means of three experiments. *, *p* < 0.05. IL-6 silencing vectors significantly sensitized tumor cells to irradiation. **b**. Effects of IL-6 on *in vivo* radiosensitivity, as assessed by evaluation of tumor growth following irradiation (15 Gy). The y-axis shows the tumor volume ratio at each time point, divided by the tumor volume at irradiation. (C-V, cells transfected with the control vector; IL-6 SV, cells transfected with the IL-6 silencing vector). **c**. The *in vitro *effects of IL-6 inhibition on p-STAT3 and p-H2AX levels and cell death, as evaluated by IHC staining and Annexin V-PI staining in irradiated cells. **d**. An IL-6-silencing vector decreased angiogenesis in tumors, as evaluated by immunoblotting and IHC analysis of tumor specimens 14 days after local irradiation. Representative slides are shown. The Y axis represents the ratio normalized by the value of target protein in tumor transfected with control vectors. Columns, means of 3 separate experiments; bars, SD. *, *P* < 0.05. (C-V, cells transfected with the control vector; IL-6 SV, cells transfected with the IL-6 silencing vector).

Regarding clinical data, the expression of IL-6 was significantly associated with a lower rate of response to curative treatment in the 173 esophageal SCC patients (Table 1), and a lower pathological complete response rate in the 55 patients who underwent surgical intervention (Table 3). Therefore, we investigated the role of IL-6 in treatment resistance and the underlying mechanisms. Colony-forming assay data (Figure 5a) and the *in vivo* delay in tumor growth (Figure 5b) demonstrated that the IL-6 silencing vector significantly sensitized esophageal cancer cells to irradiation. As shown in Figure 5c-d, inhibition of IL-6 increased cell death and DNA damage and attenuated STAT3 activation after irradiation. Tumor vascularity have been shown to be linked to the tumor-bed effect induced by irradiation [22]. To investigate whether angiogenesis underlies radiation resistance triggered by IL-6, the vascular network within the tumor by MVD analysis and the expression of VEGF after irradiation were measured. As shown in Figure 5d, the IL-6 silencing vector significantly decreased angiogenesis by evidence of decreased MVD and VEGF staining in irradiated tumors.

**Table 3:** Significance of IL-6 expression stratified according to the complete response to neoadjuvant treatment in 55 patients with surgery resection

		**IL-6**		
		**+**	**-**	
Pathologic CR	**+**	3	11	*P = 0.052*
	**-**	21	20	

### Correlation between the IL-6 level and clinical outcome

**Figure 6:**
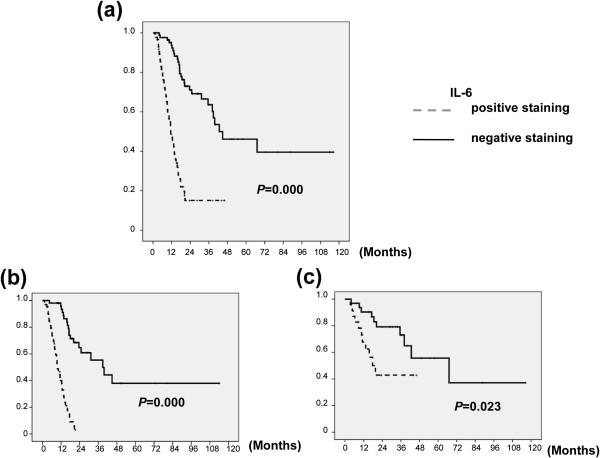
**Correlation between IL-6 level and clinical outcome. **Survival differences according to IL-6 positivity for (**a**) all 173 patients, (**b**) patients treated with CCRT and (**c**) patients who underwent pre-operative CCRT and surgery. The IL-6-positive group exhibited shorter survival than the IL-6-negative group.

As shown in Figure 6, IL-6 is a significant predictor for OS. The median OS times were 16 and 42 months in patients who completed CCRT treatment and those who underwent surgical intervention, respectively. In addition, Tables 1, 2, 3 showed that IL-6 was significantly correlated with a greater probability of developing distant metastasis and a higher recurrence rate after curative treatment. In univariate and multivariate analyses, poor treatment response, no tumor resection, and positive IL-6 staining were significantly associated with shorter survival (Tables 4, 5, 6). Furthermore, in the subgroup of 118 patients treated with CCRT, but not in the 55 who underwent surgical intervention, positive IL-6 staining retained predictive power concerning survival in a multivariate analysis (Tables 7, 8).

**Table 4:** Univariate analysis to determine factors associated with prognosis

**Variables**	**P value for overall survival**	**P value for disease-free survival**
Clinical stage	0.271	0.006*
Tumor resection	0.001*	0.000*
Positive staining for IL-6	0.000*	0.000*
Positive staining for p-STAT3	0.000*	0.000*
Local-regional Recurrence	0.002*	0.000*
Distant metastasis	0.03*	0.000*
Treatment response	0.000*	0.000*

**Table 5:** Multivariate analysis to determine molecular markers associated with prognosis (OS) of patients

**Variables**	**Odd ratios**	**95% confidence interval**	**p**
IL-6 staining	5.319	2.906-9.734	<0.001*
p-STAT3 staining	0.926	0.529-1.593	0.761
Tumor resection	0.454	0.270-0.762	0.003*
Clinical stage	0.908	0.531-1.551	0.723
Treatment response	2.601	1.583-4.276	<0.001*
Recurrence and/or distant metastasis	4.172	1.700-10.240	0.002*

**Table 6:** Multivariate analysis to determine molecular markers associated with prognosis (DFS) of patients

**Variables**	**Odd ratios**	**95% confidence interval**	**p**
IL-6 staining	5.424	3.349-8.784	<0.001*
p-STAT3 staining	0.724	0.465-1.128	0.153
Tumor resection	0.365	0.241-0.553	<0.001*
Clinical stage	1.655	1.058-2.589	0.027*
Treatment response	3.719	2.135-4.733	<0.001*

**Table 7:** Multivariate analysis to determine factors associated with prognosis (OS) of patients with definite CCRT treatment

**Variables**	**Odd ratios**	**95% confidence interval**	**p**
IL-6 staining	7.622	3.580-16.226	<0.001*
p-STAT3 staining	1.032	0.524-2.031	0.928
Clinical stage	0.828	0.409-1.677	0.600
Treatment response	2.327	1.314-4.120	0.004*
Recurrence and/or distant metastasis	4.576	1.292-16.199	0.018*

**Table 8:** Multivariate analysis to determine factors associated with prognosis (OS) of patients with pre-op CCRT and surgery

**Variables**	**Odd ratios**	**95% confidence interval**	**p**
IL-6 staining	1.738	0.537-5.622	0.356
p-STAT3 staining	1.152	0.364-3.643	0.810
Clinical stage	0.860	0.332-2.232	0.757
Pathologic CR	1.883	0.518-6.842	0.336
Recurrence and/or distant metastasis	4.205	1.138-15.538	0.031*

## Discussion

Understanding the molecular mechanisms underlying aggressive tumor growth in esophageal SCC is pivotal to identifying novel targets for pharmacological intervention. Furthermore, clinical data [2,3,23] suggest the need for markers that predict responses to neoadjuvant CCRT and help to identify patients at high risk of tumor recurrence and distant metastasis. However, no suitable markers have been identified to date. The inflammatory cytokine IL-6 contributes to the growth and progression of various malignancies, such as HNSCC, prostate cancer and gastrointestinal cancer, and acts as an autocrine growth factor and an anti-apoptotic factor for various stimuli, including anticancer agents [11,24-27]. Moreover, the IL-6/STAT3 pathway is important for the development of inflammation-associated intestinal tumorigenesis. Similar to other series [11,28], we found that IL-6 and IL-6R were detected in both esophageal cancer specimens and esophageal carcinoma cell lines. IL-6 expression was higher in esophageal cancer specimens compared with non-malignant tissues. Furthermore, positive staining for IL-6 was associated with higher tumor stage, higher rates of tumor recurrence and distant metastasis. To further investigate whether IL-6 was responsible for aggressive tumor growth in esophageal SCC, we suppressed IL-6 in esophageal cancer cells using a silencing vector. Data obtained from cell and xenograft tumor growth experiments revealed that inhibiting IL-6 resulted in slower tumor growth and reduced invasiveness. The transformation of an epithelial cell into a mesenchymal cell appears to be functionally relevant to the invasive characteristics of epithelial tumors, including esophageal cancer [19,29,30]. At the molecular marker level, EMT is characterized by the loss of E-cadherin and increased expression of invasion-related factors [31]. In cell experiments, the IL-6 silencing vector induced both an increase in E-cadherin levels in esophageal cancer cells, and decreases in MMP-9 levels. Constitutive activation of STAT3 signaling has been reported to contribute to oncogenesis by promoting proliferation and EMT, and IL-6 is a major activator of JAK/STAT3 signaling [8,32-34]. Therefore, we examined the links between STAT3 activation, IL-6 and EMT. When blocking STAT3 activation by STAT3 siRNA or JAK inhibitor, the decreases in EMT-related protein levels were comparable to those induced by IL-6 silencing vector. Furthermore, IHC data obtained from clinical samples demonstrated that IL-6 expression was significantly correlated with p-STAT3 staining. Therefore, it is likely that STAT3 activation plays a role in transmitting IL-6 signals to downstream targets that regulate EMT and invasiveness.

In the present study, IL-6 silencing vectors significantly decreased IL-6 levels seen in the supernatant of cell culture medium and the serum of mice bearing tumors. Moreover, in the clinic, IL-6 serum levels seem to be elevated in a subgroup of patients with local-regional failure or distant metastasis. We assume that circulating IL-6 plays an important role to stimulate tumor growth *in vivo*, besides direct action on malignant cells. Angiogenesis is one of the mechanisms that promote tumor progression, and CD31-mediated endothelial cell-cell interactions are involved in angiogenesis. Moreover, STAT3 activation has been reported to modulate the expression of genes that mediate angiogenesis; *e*.*g*., VEGF [35]. Accordingly, the links between IL-6, angiogenesis, and promotion of cancer in tumor-bearing mice were further investigated using a xenograft tumor model. Our data from animal experiments demonstrated that IL-6 level positively linked with angiogenesis in addition to EMT. These findings indicated that the promotion of EMT changes and angiogenesis mediate the aggressive tumor growth noted in IL-6 expressing esophageal cancer, at least in part.

Radiotherapy is a well-established therapeutic modality in oncology. It provides survival benefits in several cancer types, including esophageal cancer. For esophageal SCC, treatment response is an independent prognostic factor. We demonstrated that positive IL-6 staining was significantly associated with a lower response rate after treatment in patients with esophageal SCC. As determined using clonogenic assays and delayed tumor growth, inhibition of IL-6 by transfection of an IL-6 silencing vector increased radiosensitivity. Extensive DNA damage caused by radiation and anti-cancer agents can result in cell death or sensitivity to clinical treatment if left unrepaired [36]. Phosphorylated H2AX is an indicator of double-strand breaks, and its expression after irradiation correlates with treatment sensitivity [37]. Our data demonstrate that inhibition of IL-6 was associated with increased radiation-induced DNA damage and increased cell death. Moreover, the data from xenograft tumors demonstrated that inhibition of IL-6 attenuated angiogenesis and decreased p-STAT3 activation after irradiation. The expression of angiogenic factors is suggested to have predictive value for treatment response and outcome in patients with esophageal cancer [38,39]. Therefore, we suggest activation of STAT3 signaling and angiogenesis after irradiation was reported to be responsible for resistance to treatment and tumor regrowth after irradiation triggered by IL-6.

We further examined the predictive power of IL-6 for prognosis in esophageal SCC. Besides a lower response to treatment, enhanced expression of IL-6 was significantly associated with a higher clinical stage, a greater risk of distant metastasis and shorter survival. In a multivariate analysis, IL-6 retained predictive power for OS.

These findings indicate that IL-6-positive esophageal cancer provides a suitable microenvironment for the development of tumor growth and treatment resistance mediated by induction of angiogenesis, enhancement of cell mobility, and promotion of EMT. Our data support the emerging notion that IL-6 and p-STAT3 are clinically significant predictors, and suggest that IL-6 may represent a suitable target for esophageal SCC treatment.

## Competing interests

The authors confirm that there are no conflicts of interest that could be perceived as prejudicing the impartiality of the research reported.

## Authors’ contributions

MFC conceived of the study, performed the study and coordination and assisted in editing of manuscript. PTC performed the study and drafted the manuscript. MSL conceived of the study and participated in its design and coordination. PYL helped in histology and IHC staining. WCC conceived part of the study and performed the statistical analysis. KDL participated in its design and coordination. All authors read and approved the final manuscript.

## Supplementary Material

Additional file 1 Supplementary methods. Click here for file
