# Supplementary material for: IL-6 expression predicts treatment response and outcome in squamous cell carcinoma of the esophagus
Source: Mol Cancer. 2013 Apr 5;12:26. doi: 10.1186/1476-4598-12-26 (PMC3667147; doi:10.1186/1476-4598-12-26)
Supplement: Additional file 1 — Supplementary methods. [file 1476-4598-12-26-S1.doc]

**Supplementary methods**

*Immunohistochemical staining (IHC)*

Formalin-fixed, paraffin-embedded tissues were cut into 4-μm sections, mounted on slides, deparaffinized with xylene and dehydrated using a graded ethanol series. Antigen retrieval, with the use of citric acid (pH 6.0) at 97°C for 30 minutes, was followed by treatment with3% hydrogen peroxide. The slides were incubated overnight at 4°C with antibodies against specific proteins. Antibodies specific for p-H2AX, MMP-9, vascular endothelial growth factor (VEGF), CD31 and IL-6 were obtained from Santa Cruz Biotechnology, Inc. (Santa Cruz, CA), Chemicon (Temecula, CA) and Research & Diagnostics Systems, Inc. (Minneapolis, MN USA), respectively. They were used at 1:100 dilutions. Anti-pSTAT3^Tyr705^ purchased from Cell Signaling (Danvers, MA) was used at a 1:50 dilution. After three washes in phosphate-buffered saline (PBS), the sections were incubated with biotinylated secondary antibody for10 min and stained with peroxidase-avidin and washed again in PBS; then 3-amino-9-ethylcarbazole solution was added. The sections were counterstained with hematoxylin. The IHC data were analyzed using Image Pro Plus 6.3 (IPP). Microvascular density (MVD) measurements were using CD31 staining simultaneously obtained within each area at ×100 magnification. Each immunoreactive endothelial cell cluster in contact with the selected field was counted as an individual vessel. The specimens were assessed using the semi-quantitative immunoreactive score (IRS). The IRS was calculated by multiplying the staining intensity (0 = no staining, 1 = weak staining, 2 = moderate staining, and 3 = strong staining) by the percentage of positively stained cells (0 = less than 10% of cells stained, 1 = 11–50% of cells stained, 2 = 51–80% of cells stained, and 3 = more than 81% of cells stained). The criterion for positive staining was an IRS score of ≥2.

*Tumor xenografts*

Eight-week-old male athymic nude mice were used and all animal experiments conformed to the protocols approved by the experimental animal committee of our hospital. CE81T cancer cell transfectants (1×10^6^ per implantation, five animals per group) were subcutaneously implanted in the dorsal gluteal region. Tumor size was measured every 3 days after implantation (day 0), and tumor volume was calculated assuming an ellipsoid shape. The effects of IL-6 on tumor growth were investigated *in vivo* by analyzing the tumor growth curves for each cell type. To determine *in vivo* radiosensitivity, local irradiation (15 Gy) was performed when the tumor volume reached 500 mm^3^, and tumor size was measured every 3 days thereafter. For local irradiation, anesthetized mice were restrained, and irradiated to bilateral posterior legs containing ectopic tumors using X-rays (6 MV) from a linear accelerator with a 1.5-cm bolus on the surface. Control mice were subjected to sham irradiation. The curve of tumor growth in mice subjected to irradiation was determined by relative tumor volume normalized to the tumor size at the time of irradiation. Radiosensitivity was indicated by a growth delay (*i*.*e*., the time required for the tumor to recover its previous volume after irradiation). Duplicate experiments were performed for growth delay analyses.

*Immunoblotting*

Cells were treated with lysis buffer (Calbiochem, La Jolla, CA). An NE-PER kit (Pierce, Rockford, IL) was used to separate nuclear and cytoplasmic proteins. The equal amount of protein was loaded in a 4–20% gradient SDS–PAGE gel, the protein was transferred onto nitrocellulose filters after separated in the gel. Blots were blocked in 2% BSA in TBST for 1 h, the membranes were incubated overnight (4°C) with antibodies against IL-6, IL-6 receptor (IL-6R), VEGF, E-cadherin, STAT3, pSTAT3^Tyr705^, pH2AX and MMP-9 purchased from Santa Cruz Biotechnology, Inc., R & D Systems and Cell Signaling (Danvers, MA). The membranes were incubated with HRP conjugated secondary anti-goat, anti-mouse, or anti-rabbit antibodies (dilution 1:1,000–1:2,000), respectively, for 1 h at room temperature. Proteins were visualized by ECL, exposed to an X-ray film and developed with a X-ray processor. The membranes were reprobed with an antibody against r-tubulin or nuclear lamin to normalize protein loading.

*Immunofluorescence staining (IF)*

Cells demonstrating exponential growth were seeded onto cover slips for immunofluorescence staining with or without treatment. At the indicated times after treatment, cells were fixed, permeabilized with 2% paraformaldehyde for 5 min and washed with PBST. The slides were incubated for 1 h at room temperature with antibodies against E-cadherin, IL-6 and p-STAT3, and for 1 h with a Texas Red-conjugated secondary antibody. The slides were counterstained with DAPI to visualize nuclei. After two washes with PBST, specific target proteins were visualized using a fluorescence microscope.

Enzyme-linked immunosorbent assay analysis of IL-6 levels *in vitro and in vivo*

Levels of IL-6 in cell supernatants and murine serum samples were analyzed using an IL-6 Quantikine ELISA Kit (R&D system). To measure IL-6 levels in cellular supernatants, cells were cultured in 1 ml of serum-free medium for 24 h in six-well plates. The medium was collected and clarified by centrifugation at 3,000 × g. To measure circulating IL-6 levels in vivo, blood was removed from the heart and serum. The samples were stored frozen before being subjected to IL-6 assay.

*Statistical analysis*

The significance of differences between samples was determined using Student’s t-test. Data are presented as the means ± standard error of the mean (SD). All experiments were carried out at least twice, independently, and each comprised three replicates. A probability level of *p*<0.05 was adopted throughout to determine statistical significance, unless otherwise stated.
